# Protein-bound uremic toxins are associated with cognitive function among patients undergoing maintenance hemodialysis

**DOI:** 10.1038/s41598-019-57004-7

**Published:** 2019-12-31

**Authors:** Yi-Ting Lin, Ping-Hsun Wu, Shih-Shin Liang, Mwenya Mubanga, Yuan-Han Yang, Ya-Ling Hsu, Mei-Chuan Kuo, Shang-Jyh Hwang, Po-Lin Kuo

**Affiliations:** 1 Graduate Institute of Clinical Medicine, College of Medicine, Kaohsiung Medical University, Kaohsiung, Taiwan; 2 Faculty of Medicine, College of Medicine, Kaohsiung Medical University, Kaohsiung, Taiwan; 3 Department of Biotechnology, College of Life Science, Kaohsiung Medical University, Kaohsiung, Taiwan; 4 Department of Medical Sciences, Molecular Epidemiology, Uppsala University, Uppsala, Sweden; 5 Department of Family Medicine, Kaohsiung Medical University Hospital, Kaohsiung, Taiwan; 6 Department of Neurology, Kaohsiung Municipal Ta-Tung Hospital, Kaohsiung Medical University, Kaohsiung, Taiwan; 7 Graduate Institute of Medicine, College of Medicine, Kaohsiung Medical University, Kaohsiung, Taiwan; 8 Division of Nephrology, Department of Internal Medicine, Kaohsiung Medical University Hospital, Kaohsiung, Taiwan; 9 Institute of Population Sciences, National Health Research Institutes, Miaoli, Taiwan

**Keywords:** Mass spectrometry, Outcomes research, Haemodialysis, Dementia

## Abstract

Patients with chronic kidney disease have a greater risk of cognitive impairment. Cerebral uremic solute accumulation causes uremic encephalopathy; however, the association of protein-bound uremic toxins on cognitive function remains unclear. The present study aimed to investigate the association of two protein-bound uremic toxins, namely indoxyl sulfate (IS) and p-cresyl sulfate (PCS), on cognitive function in patients receiving hemodialysis (HD) for at least 90 days. Circulating free form IS and PCS were quantified by liquid chromatography/mass spectrometry. Mini-Mental State Examination (MMSE) and Cognitive Abilities Screening Instrument (CASI) were used to evaluate cognitive function. In total, 260 HD patients were recruited with a mean age of 58.1 ± 11.3 years, of which, 53.8% were men, 40% had diabetes, and 75.4% had hypertension. The analysis revealed that both free IS and free PCS were negatively associated with the CASI score and MMSE. After controlling for confounders, circulating free IS levels persisted to be negatively associated with MMSE scores [β = −0.62, 95% confidence interval (CI): −1.16 to −0.08] and CASI scores (β = −1.97, 95% CI: −3.78 to −0.16), mainly in the CASI domains of long-term memory, mental manipulation, language ability, and spatial construction. However, there was no correlation between free PCS and total MMSE or total CASI scores after controlling for confounders. In conclusion, circulating free form IS, but not PCS is associated with lower cognitive function test scores in HD patients. Thus, a further study is needed to evaluate whether a decrease in free IS levels can slow down cognitive decline in HD patients.

## Introduction

Dementia is characterized by progressive loss of daily life functions and the development of psychiatric symptoms. Patients with chronic kidney disease (CKD) or end-stage kidney disease (ESKD) have a higher rate of cognitive impairment, with approximately threefold higher dementia prevalence than similar aged individuals in the general population^1^. Dementia further worsens the adverse outcomes, leading to mortality, hospitalization, disability, and dialysis withdrawal^2,3^. Patients with CKD not only have the same risk factors for dementia as the general population^4^, but also have additional nephrogenic risk factors^4,5^. Previous studies have addressed the role of cerebrovascular disease, anemia, and hyperparathyroidism in dementia development in patients with CKD or ESKD^4,5^, but the association of uremic toxins on cognitive function remains unclear. The accumulation of uremic solutes in the blood and the brain may affect cognitive function^6^. Uremic toxins are classified into three groups based on their protein binding property and molecular weight: protein-bound solutes, middle molecules, and small water-soluble solutes^7^. Although hemodialysis (HD) eliminates small water-soluble uremic toxins, the remaining middle-molecule and protein-bound uremic toxins interfere with cognitive function by disrupting the brain barrier^8^. Similarly, peritoneal dialysis clearance of protein-bound uremic toxins is lower than the clearance of small solutes^9–12^, with residual renal function accounting for most protein-bound uremic toxin removal in peritoneal dialysis patients^9,10^.

Indoxyl sulfate (IS) and p-cresyl sulfate (PCS), known as protein-bound uremic toxins, are difficult to eliminate via dialysis because they tightly bind to albumin in the blood; consequently, the IS level in brain and plasma in patients with CKD is high compared with control subjects^13^. IS may trigger cerebral dysfunction through the induction of inflammation and oxidative stress, which are initiated by astrocytes and microglia^14^, further reducing neuronal viability and contributing to neurodegeneration^15^. However, only a few studies have evaluated the association between protein-bound uremic solute levels and cognitive impairment, and the results were inconclusive^16,17^. Therefore, we investigated the association of IS and PCS with cognitive function in HD patients.

## Methods

### Bioinformatics of blood-brain barrier prediction

The brain is safeguarded from exogenous compounds through the blood-brain barrier (BBB). The potential of a toxin to enter the brain is an essential parameter when considering the toxicity, therefore, a BBB predictor was designed to determine whether a compound can cross the BBB. *In silico* data, the prediction was analyzed by the online BBB server (http://www.cbligand.org/BBB/predictor.php) using the admetSAR database^18^. The BBB predictor predicts the ability of a compound to pass the BBB and was built using support vector machine algorithms for the four types of fingerprints (Molecular ACCess System fingerprints, Open babel fingerprint 2, Molprint 2D, and PubChem fingerprint) of 1593 reported compounds^19^. Absorption, Distribution, Metabolism, and Toxicity (ADMET) properties were calculated based on the admetSAR database that offers various chemical data with known ADMET properties.

### Case subjects in HD cohort

**Figure 1:**
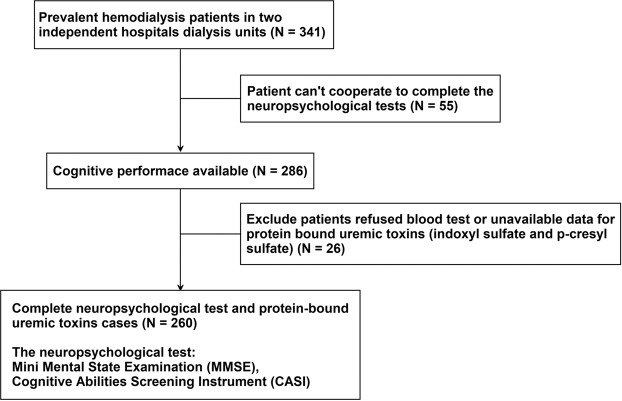
Flow chart showing the study design.

From August 2016 to January 2017, patients were recruited at Kaohsiung Medical University Hospital to establish the HD cohort. Participants with maintenance dialysis for more than 90 days (N = 341) were eligible. In total, 286 individuals were screened using neuropsychological tests. Participants who refused to undergo blood tests or had missing data regarding protein-bound uremic toxin (IS and PCS) measurement (N = 26) were excluded from the final analysis (Fig. 1). All patients received regular HD three times per week through automated volumetric machines, with each HD session using high-efficiency dialyzers and lasting for 4 hours. The dialysate flow was maintained at 500 mL/min, the blood flow rate was maintained at 250 and 300 mL/min, and single-pool Kt/V was more than 1.2 per week. Informed consent was acquired from all subjects. Ethical approval was granted by the Institutional Review Board of Kaohsiung Medical University (KMUHIRB-E(I)-20160095). All experiments were performed in accordance with relevant guidelines and regulations.

### Control subjects of the general population

A voluntary non-dialysis comparison group of 55 independently living subjects was recruited by advertisement in the community. Eligibility criteria for the comparison group were the same as for the HD subjects, with additional exclusions of diagnosed ESKD or CKD. Similar to the HD patients, circulating free form IS and PCS were measured and neuropsychiatric tests (MMSE, CASI, and CES-D) were also conducted.

### Comorbidity and clinical laboratory data measurement

For all participants, demographic data (age, sex, and education level), dialysis duration, medical history, and biochemical data were extracted from routine electronic healthcare records. Hypertension was defined as blood pressure ≥140/90 mmHg or antihypertensive drug use; diabetes was defined as HbA1C ≥6.5% or antidiabetic drug use. Participants’ history of coronary artery disease and cerebrovascular disease was obtained based on physicians’ diagnosis. Blood samples of patients were obtained at a single midweek dialysis session after overnight fasting through vascular access before their scheduled HD session. Biochemical data of HD patients were obtained at the beginning of the HD session within 30 days before cognitive testing.

### Cognitive function assessment

Two tests were used in the current study to evaluate cognitive function by two well-trained psychologists: Mini-Mental State Examination (MMSE)^20,21^ and Cognitive Abilities Screening Instrument (CASI)^22,23^ (Supplementary Table 1). The Center for Epidemiological Studies Depression (CES-D) with scores ranging from 0 to 30 was applied to determine the depression symptoms score, as part of the broader neurocognitive assessment.

### Measurement of circulating free form indoxyl sulfate and p-cresyl sulfate levels

For free form PCS and IS free analyses, each human serum samples (300 μL) was packed into a centrifugal filter device (Amico Ultra 3 K, MerckMillipore) and centrifuged for 30 min at 13,300 g at 4°C. Then, the supernatant was carefully transferred into another Eppendorf tube and evaporated with a spin vacuum instrument. The lyophilized samples were re-dissolved with 100 µL 30% acetonitrile (MeCN) with 0.1% formic acid, then 100 µL of the solution was added 10 µL IS-d4 (internal standard purchased from Sigma-Aldrich, 1000 ng/mL) and filtrated with 0.22 µm polytetrafluoroethylene (PTFE) filters for mass spectrometer analysis. The tandem mass spectrometry system was equipped with micro electrospray ionization (ESI) ion source, coupled with an Acella 1250 Ultra-high-performance liquid chromatography (UHPLC) analytical system (Thermo Fisher Scientific Inc., Waltham, MA, USA) with 3.0 kV setting. The samples were separated using a Shiseido HPLC CAPCELL PAK C18 MGII column (150 mm × 1.5 mm, 3.0 µm; Tokyo, Japan) after injection into the UHPLC system via the Acella 1250 autosampler. The mobile phases contained 0.1% (v/v) FA (A) in water and 0.1% (v/v) FA (B) in acetonitrile with a 250 mL/min flow rate. The linear gradient was set as follows: 30%, 30–60%, 60–98%, 98%, 98–30%, 30% in 2/6/3/2/0.1/6.9 minutes respectively (B). The negative ion mode with a voltage of 2.5 kV was set up as detection mode of the mass spectrometer, the temperature for the vaporizing and capillary temperatures was 300 °C and 350 °C, and the pressure setting for sheath gas and aux gas were 35 and 10 with a collision pressure of 1.5 and collision energy of 22 V. The quantification was evaluated by survey scan mode multiple reaction monitoring (MRM) transitions 212 > 80 and 212 > 132 for IS and 187 > 80 and 187 > 107 for PCS, using Xcalibur software (version 2.2, Thermo-Finnigan Inc., San Jose, CA, USA).

### Statistical analysis

Data are shown as mean ± standard deviation or percentages as appropriate. The association of cognitive function test scores with IS or PCS was evaluated using linear regression models controlling for age, sex, and education level. To identify the independent determinants of IS and PCS, multivariate linear regression analysis with forward stepwise selection of variables was performed to assess the relationship between uremic toxins levels and MMSE/CASI scores, with *p* values set at 0.2 in the models for independent variables to enter and stay, subsequently *p* values < 0.05 in the final elimination step. The covariates adjusted for in the model were age, sex, education level, depression scale (CES-D), comorbidities (hypertension, diabetes mellitus, cerebrovascular disease, and coronary artery disease), clinical data (systolic and diastolic blood pressure, hemoglobin, Kt/V, and blood urea nitrogen), and HD duration. The results are reported as the β coefficient with a 95% confidence interval (CI). In addition, ROC-derived AUC cutoff values were used as cutoffs for statistical analyses rather than using an arbitrary cutoff level. The results are reported as the odds ratio (OR) with 95% CI. Two-tailed *p* < 0.05 was considered statistically significant. STATA version 15 and SAS version 9.4 were used to perform statistical analyses.

## Results

### Permeability prediction through the blood-brain barrier for indoxyl sulfate and p-cresyl sulfate

Using *in silico* simulation analysis, the BBB prediction score was applied to investigate the BBB penetration ability of IS or PCS. If the BBB score is >0, the compound can be considered as BBB+. The BBB predictor classified IS and PCS as BBB+ compounds (Supplementary Fig. 1). Furthermore, the BBB penetration probability was 0.8727 and 0.9405 for IS and PCS according to ADMET prediction, indicating that IS and PCS can cross the BBB.

### Demographic and clinical characteristics

In total, 260 participants in the HD cohort and 55 control participants received baseline cognitive evaluation. Participants with HD are younger and have more comorbidities. The mean age of the HD participants was 58.1 ± 11.3 years, 53.8% were men, 40% had diabetes, and 75.4% had hypertension. In addition, 21.5% of participants had coronary artery disease, and 11.2% had cerebrovascular disease. The mean HD duration was 7.05 ± 5.83 years.

The HD patients had higher circulating IS and PCS levels than the control group significantly (1.34 ± 0.95 μg/mL for IS and 1.06 ± 0.97 μg/mL for PCS in HD patients versus 0.01 ± 0.04 μg/mL for IS and 0.03 ± 0.04 μg/mL for PCS in control subjects) (Table 1 and Supplementary Table 2). Furthermore, the mean cognitive function test scores of the HD group were lower than the control groups significantly (24.28 ± 4.73 for MMSE and 80.49 ± 16.69 for CASI in HD patients versus 26.91 ± 1.69 for MMSE and 90.46 ± 6.15 for CASI in control subjects) (Table 1 and Supplementary Table 2).

**Table 1:** Baseline clinical parameters.

Participants (N = 260)	
Age (years)	58.1 ± 11.3
Female	120 (46.2%)
**Education**	
College	63 (24.2%)
Senior high school	77 (29.6%)
Junior high school	56 (21.5%)
Elementary school	50 (19.2%)
No	12 (4.6%)
Hemodialysis duration (years)	7.05 ± 5.83
Systolic blood pressure	145.54 ± 26.93
Diastolic blood pressure	79.28 ± 14.91
**Comorbidities**	
Diabetes mellitus	104 (40.0%)
Hypertension	196 (75.4%)
Coronary artery disease	56 (21.5%)
Cerebrovascular disease	29 (11.2%)
**Laboratory data**	
Hemoglobin	10.72 ± 1.23
Blood urea nitrogen	65.09 ± 13.90
Total Kt/V	1.57 ± 0.24
**Uremic toxins**	
Free form Indoxyl sulfate (μg/ml)	1.34 ± 0.95
Free form p-cresyl sulfate (μg/ml)	1.06 ± 0.97
**Neuropsychiatric test**	
Mini–Mental State Examination	24.28 ± 4.73
Cognitive Abilities Screening Instrument	80.49 ± 16.69
Long term Memory	9.48 ± 1.55
Short term Memory	8.02 ± 2.96
Attention	6.77 ± 1.76
Mental manipulation	7.78 ± 2.54
Orientation	16.19 ± 3.71
Abstract thinking	7.94 ± 2.89
Language	9.12 ± 1.73
Spatial construction	7.85 ± 3.01
Name fluency	7.36 ± 2.58
Center for Epidemiological Studies Depression	11.04 ± 7.34

### Associations of circulating free form IS and PCS levels and cognitive function

We evaluated the association between protein-bound uremic toxins and cognitive function test scores (MMSE, CASI, and CASI cognitive evaluation domains) in HD participants using a regression model. IS was negatively correlated with the scores of MMSE, CASI, and CASI subdomains of long-term memory, mental manipulation, orientation, abstract thinking, language, and spatial construction (Table 2, model 1). PCS negatively correlated with the scores of MMSE, CASI and CASI subdomains of long-term memory, short-term memory, attention, orientation, language and spatial construction (Table 3, model 1).

**Table 2:** Association between circulating free form indoxyl sulfate levels and cognitive function test scores in hemodialysis participants using linear regression analysis without and with adjusted for confounders.

Cognitive test	Model 1		Model 2	
	β coefficient (95% CI)	*p* value	β coefficient (95% CI)	*p* value
Mini-Mental State Examination	−0.97 (−1.56 to −0.37)	0.001	−0.62 (−1.16 to −0.08)	0.023
Cognitive Abilities Screening Instrument	−3.43 (−5.53 to −1.33)	0.001	−1.97 (−3.78 to −0.16)	0.033
Long term memory	−0.22 (−0.42 to −0.02)	0.030	−0.20 (−0.39 to −0.01)	0.036
Short term memory	−0.38 (−0.75 to 0.00)	0.050	−0.13 (−0.47 to 0.21)	0.442
Attention	−0.19 (−0.41 to 0.04)	0.104	−0.10 (−0.31 to 0.11)	0.343
Mental manipulation	−0.52 (−0.84 to −0.20)	0.001	−0.36 (−0.65 to −0.06)	0.018
Orientation	−0.61 (−1.08 to −0.14)	0.011	−0.41 (−0.87 to 0.04)	0.076
Abstract thinking	−0.45 (−0.82 to −0.09)	0.016	−0.19 (−0.51 to 0.14)	0.264
Language	−0.35 (−0.56 to −0.13)	0.002	−0.25 (−0.45 to −0.04)	0.018
Spatial construction	−0.53 (−0.92 to −0.15)	0.006	−0.38 (−0.73 to −0.03)	0.034
Name fluency	−0.19 (−0.51 to 0.14)	0.270	0.05 (−0.25 to 0.36)	0.728

Model 1: crude model.

Model 2: adjusted model with controlling for age, sex, education level, depression scale, and comorbidities (diabetes mellitus, hypertension, coronary artery disease, and cerebrovascular disease), hemoglobin, blood urea nitrogen, Kt/V, hemodialysis duration.

**Table 3:** Association between circulating free form p-cresyl sulfate levels and cognitive function test scores in hemodialysis participants using linear regression analysis without and with adjusted for confounders.

Cognitive test	Model 1		Model 2	
	β coefficient (95% CI)	*p* value	β coefficient (95% CI)	*p* value
Mini-Mental State Examination	−0.79 (−1.37 to −0.21)	0.008	−0.44 (−0.97 to 0.08)	0.100
Cognitive Abilities Screening Instrument	−2.97 (−5.03 to −0.91)	0.005	−1.71 (−3.48 to 0.06)	0.058
Long term memory	−0.21 (−0.40 to −0.01)	0.036	−0.17 (−0.36 to 0.01)	0.072
Short term memory	−0.38 (−0.74 to −0.01)	0.045	−0.19 (−0.52 to 0.14)	0.256
Attention	−0.23 (−0.45 to −0.01)	0.040	−0.12 (−0.33 to 0.08)	0.245
Mental manipulation	−0.21 (−0.53 to 0.10)	0.188	−0.05 (−0.34 to 0.24)	0.729
Orientation	−0.49 (−0.95 to −0.03)	0.036	−0.33 (−0.78 to 0.12)	0.146
Abstract thinking	−0.32 (−0.68 to 0.04)	0.082	−0.15 (−0.47 to 0.17)	0.371
Language	−0.33 (−0.54 to −0.11)	0.003	−0.22 (−0.42 to −0.02)	0.035
Spatial construction	−0.56 (−0.93 to −0.19)	0.003	−0.38 (−0.73 to −0.04)	0.029
Name fluency	−0.25 (−0.57 to 0.08)	0.134	−0.10 (−0.39 to 0.20)	0.522

Model 1: crude model.

Model 2: adjusted model with controlling for age, sex, education level, depression scale, and comorbidities (diabetes mellitus, hypertension, coronary artery disease, and cerebrovascular disease), hemoglobin, blood urea nitrogen, Kt/V, hemodialysis duration.

Using multivariate regression model controlling for confounders (age, sex, education level, depression scale, comorbidities, hemoglobin, blood urea nitrogen, Kt/V, and hemodialysis duration), circulating free form IS levels were persistently negatively associated with MMSE scores (β = −0.62, 95% CI: −1.16 to −0.08, *p* = 0.023), CASI scores (β = −1.97, 95% CI: −3.78 to −0.16, *p* = 0.033), and the scores of CASI domains of long-term memory (β = −0.20, 95% CI: −0.39 to −0.01, *p* = 0.036), mental manipulation (β = −0.36, 95% CI: −0.65 to −0.06, *p* = 0.018), language ability (β = −0.25, 95% CI: −0.45 to −0.04, *p* = 0.018), and spatial construction (β = −0.38, 95% CI: −0.73 to −0.03, *p* = 0.034) (Table 2, model 2). Free form PCS was only negatively associated with language ability (β = −0.22, 95% CI: −0.42 to −0.02, *p* = 0.035) and spatial construction (β = −0.38, 95% CI: −0.73 to −0.04, *p* = 0.029) in the multivariate linear regression analysis. (Table 3, model 2). However, there was no significant association between free form PCS and total MMSE or CASI score (Table 3, model 2).

The cutoff value of IS on cognitive impairment was analyzed by ROC curves and was 1.45 μg/ml. Higher circulating IS levels (>1.45 μg/ml) were associated with cognitive impairment compared to a lower IS level in the unadjusted logistic regression model (OR, 1.97; 95% CI, 1.17 to 3.33; *p* = 0.01) and in age and sex-adjusted logistic regression model (OR, 1.75, 95% CI, 1.01 to 3.04, *p* = 0.048).

## Discussion

This study clarified the association of protein-bound uremic toxins on cognitive function in HD patients. In our study, *in silico* data predicted that IS and PCS could penetrate the BBB. The efflux of uremic toxin from the brain depends on BBB organic anion transporter^24^. However, according to the clinical study, circulating free form IS, but not PCS, was associated with impaired cognitive function. An inverse association was observed between circulating free form IS levels and the scores of MMSE, CASI, and CASI subdomains of long-term memory, mental manipulation, language ability, and spatial construction. In the fully adjusted linear regression model, circulating free form PCS levels were not significantly associated with MMSE or CASI.

Cognitive function impairment was found in HD patients compared with subjects without kidney disease in the present study and previous meta-analysis^1^. The etiologies of cognitive impairment in HD patients are multifactorial, including secondary hyperparathyroidism, cerebrovascular disease, dialysis disequilibrium, renal anemia, and uremic toxin. In a previous study, a higher IS level was associated with impaired cognitive function in patients with stage 3 CKD, but not in those with advanced CKD^16^. Furthermore, in that study, no association was observed between PCS and cognitive function, which was confirmed by our results. However, the sample size of the previous study was relatively small, particularly for patients with stage 5 CKD, which reduced the statistical power to determine the IS effect on cognitive function in patients with advanced CKD. In the present study, only ESKD patients were enrolled, so our findings provide evidence to support the negative association between circulating IS level and cognitive function in end-stage renal disease patients (HD patients). Another metabolomic study could not find an association between IS and cognitive impairment in HD patients due to the lack of power^17^, therefore, we enrolled 260 HD patients in the present study to investigate the correlation between IS and cognitive function. We evaluated the cognitive function based on MMSE and CASI to determine the association between uremic toxins and cognition. The different impact of IS and PCS on cognitive dysfunction in hemodialysis patients could be explained by the increased protein binding ability of IS which was associated with the increased free IS levels. However, the protein binding ability of PCS decreased with increasing free moiety concentrations^25^. Furthermore, it has been suggested that the affinity of PCS for human serum albumin binding is moderate at 25 °C and weak at 37 °C (the physiological temperature) using microcalorimetry analysis^26^, thus, PCS binding to albumin appears to be less strong than previously reported^26^. The higher protein binding ability is the main cause of poor removal of these protein-bound toxins by HD, therefore, these differences between IS and PCS may play a role in the observed cognitive impairment differences between IS and PCS.

Protein-bound uremic toxins, such as IS, may play a role in cognitive function decline through direct toxicity or by other putative factors, such as vascular calcification, endothelial dysfunction, oxidative stress, and inflammation^6^. HD can partially clear protein-bound uremic toxins produced by the gut microbiome^27^, depending on the affinity and conjugates to albumin. IS normally crosses the BBB via organic anion transporter 3 (OAT-3), so may accumulate in the brain due to dysfunctional OAT-3^24^. IS levels were higher in the brain and circulation among patients with CKD compared with non-CKD subjects^13^. Furthermore, increased IS levels in the blood may impair the brain transporters’ ability to remove other solutes, as IS has been shown to inhibit related transporters in kidney and liver cells^28^. Several direct pathophysiology mechanisms of IS-related neurotoxicity exist. IS stimulates the aryl hydrocarbon receptor (AhR), known as a ligand-activated transcriptional factor^29^, prolonged activations of which could cause endothelial dysfunction, thereby contributing to neurotoxicity^30,31^. Furthermore, IS direct influences glial and astrocyte function, causing neuronal damage^14^. In the presence of IS, astrocyte cells increase the release of reactive oxygen species and decrease the activation of nuclear factor-like 2 and the expression of heme oxygenase-1 and NADPH dehydrogenase quinone 1^14^. IS also induces apoptosis of human astrocytes via oxidative stress and mitogen-activated protein kinase pathway inhibition^32^. Additionally, IS may induce inflammatory and oxidative stress mediator production from glial cells, which actively contribute to various dementia forms^33^, and complex neuron-glia interaction disturbances are important in the pathophysiological mechanism for neurological disorders, including neurodegeneration^34^. Glial cell dysfunction can deteriorate neuronal function. Moreover, IS induces neuron cell death in a dose-dependent manner^14^.

AhR is expressed in the hippocampus, cerebral cortex, and cerebellum^35^. Furthermore, IS activates AhR and promotes oxidative stress in astrocytes and further implicates sensorimotor and cognitive dysfunction^35^. Considering the direct neuronal toxicity of protein-bound uremic toxins, IS may be involved in cognitive impairment in patients with CKD through vascular toxin effects and direct toxic effects on cells, especially in those without obvious cerebrovascular disease. IS increases oxidative stress leading to histological brain alterations, and inflammatory markers were found in the IS-injected mice model^14^. Moreover, IS increased inflammation and oxidative stress in primary central nervous system cells through nuclear factor-kappa B and AhR, and IS induced neuron death^15^. Furthermore, in the presence of AST-120, pro-oxidant and pro-inflammatory responses reduced in astrocytes treated with patients’ serum^15^. In an animal study, cisplatin-administered rats showed increased IS concentrations in the brain tissue, with associated increases in nephrotoxicity and disturbances in the circadian rhythm of the transcription of the clock gene rPer2^36^. The aforementioned results support the present study findings regarding IS neurotoxicity and that higher circulating levels of free form IS lead to poor cognitive function scores.

The current study is not without limitations. First, all subjects in this study received HD, not peritoneal dialysis. In peritoneal dialysis patients with significant residual renal function, more IS and PCS are removed in the urine than in the dialysate^9,10^. Thus, these findings may difficult to apply for patients with peritoneal dialysis because of the different uremic toxin concentration in patients with peritoneal dialysis^9,37^. Second, a cross-sectional study could not be used to imply causality. To gain clarity, longitudinal studies with longer follow-up durations are required. Third, to ascertain the cognitive impairment etiology, brain images were unavailable in this study. Finally, the IS concentration in the cerebrospinal fluid is possibly more crucial than circulating levels with respect to cognitive function, but this was not assessed in this study.

## Conclusion

Circulating free form IS but not PCS was associated with poor cognitive function (MMSE and CASI) in patients undergoing HD. In addition, IS was negatively associated with CASI domains of long-term memory, mental manipulation, language ability, and spatial construction. Additional studies are needed to investigate whether IS elimination in patients with advanced CKD delays cognitive decline.

## Supplementary information

Supplementary Tables and figures.
