# Supplementary material for: Protein-bound uremic toxins are associated with cognitive function among patients undergoing maintenance hemodialysis
Source: Sci Rep. 2019 Dec 31;9:20388. doi: 10.1038/s41598-019-57004-7 (PMC6938492; doi:10.1038/s41598-019-57004-7)

**Protein-bound uremic toxins were associated with cognitive function among patients undergoing maintenance hemodialysis**

Yi-Ting Lin^1,2,4,5^, Ping-Hsun Wu^1,2,8^, Shih-Shin Liang^3^, Mwenya Mubanga^4^, Yuan-Han Yang^6^, Ya-Ling Hsu^7^, Mei-Chuan Kuo^2,8^, Shang-Jyh Hwang^2,8,9^, Po-Lin Kuo^1^

^1^Graduate Institute of Clinical Medicine, College of Medicine, Kaohsiung Medical University, Kaohsiung, Taiwan

^2^Faculty of Medicine, College of Medicine, Kaohsiung Medical University, Kaohsiung, Taiwan

^3^Department of Biotechnology, College of Life Science, Kaohsiung Medical University, Kaohsiung, Taiwan

^4^Department of Medical Sciences, Molecular Epidemiology, Uppsala University, Uppsala, Sweden

^5^Department of Family Medicine, Kaohsiung Medical University Hospital, Kaohsiung, Taiwan

^6^Department of Neurology, Kaohsiung Municipal Ta-Tung Hospital, Kaohsiung Medical University, Kaohsiung, Taiwan

^7^Graduate Institute of Medicine, College of Medicine, Kaohsiung Medical University, Kaohsiung, Taiwan

^8^Division of Nephrology, Department of Internal Medicine, Kaohsiung Medical University Hospital, Kaohsiung, Taiwan

^9^Institute of Population Sciences, National Health Research Institutes, Miaoli, Taiwan

**Corresponding Authors**

Shang-Jyh Hwang, MD

Division of Nephrology, Department of Internal Medicine, Kaohsiung Medical University Hospital, Kaohsiung, Taiwan

100 Shih-Chuan 1st Road Kaohsiung 807, Taiwan

Telephone number: 886-7-3121101 ext. 7900

Fax number: 886-7-3228721

E-mail address: [sjhwang@kmu.edu.tw](mailto:sjhwang@kmu.edu.tw)

Po-Lin Kuo, PhD

Institute of Clinical Medicine, College of Medicine, Kaohsiung Medical University, Kaohsiung, Taiwan

100 Shih-Chuan 1st Road Kaohsiung 807, Taiwan

Telephone number: 886-7-3121101 ext. 2512 #33

E-mail address: kuopolin@seed.net.tw

**Supplementary Table 1.** Description of the neuropsychiatric test performed in hemodialysis patients in this study

| Neuropsychiatric test | Description |
| --- | --- |
| Cognitive Abilities Screening Instrument (CASI) | The CASI has 9 cognitive evaluation domains, including short-term memory, long-term memory, namely, language, attention, mental manipulation, orientation, list-generating fluency, drawing, and abstraction/judgment. There are total 40-items for this global cognitive test. |
| Mini-Mental State Examination (MMSE) | MMSE is the most widely used brief cognitive instrument that assesses orientation, verbal functions, memory, and construction. It is generally best suited for detecting moderate to severe deficits in dementia. |
| Center for Epidemiologic Studies Depression Scale (CES-D) | This scale is used to evaluate the frequency of depression symptoms in the past week. Scores assigned for response options are 3 for “most or all of the time,” 2 for “occasionally or a moderate amount of time,” 1 for “some or a little of the time,” and 0 for “rarely or none of the time.” |

**Supplementary Table 2.** Baseline characteristics of control subjects

| Patients ( N = 55 ) | |
| --- | --- |
| Age (years) | 60.6 ± 6.2 |
| Male | 27 ( 49.1% ) |
| Systolic blood pressure | 128.27 ± 20.59 |
| Diastolic blood pressure | 76.78 ± 11.92 |
| Education |  |
| No | 0 ( 0% ) |
| Elementary school | 5 ( 9.1% ) |
| Junior high school | 10 ( 18.2% ) |
| Senior high school | 18 ( 32.7% ) |
| College | 22 ( 40.0% ) |
| Comorbidities |  |
| Diabetes mellitus | 6 ( 10.9% ) |
| Hypertension | 16 ( 29.1% ) |
| Coronary artery disease | 0 ( 0% ) |
| Cerebrovascular disease | 0 ( 0% ) |
| Uremic toxins |  |
| Free Indoxyl sulfate (μg/ml) | 0.01 ± 0.04 |
| Free p-cresyl sulfate (μg/ml) | 0.03 ± 0.04 |
| Neuropsychiatric test |  |
| Cognitive Abilities Screening Instrument | 90.46 ± 6.15 |
| Short term Memory | 9.21 ± 2.50 |
| Long term Memory | 9.98 ± 0.13 |
| Mental manipulation | 9.2 ± 1.35 |
| Attention | 7.64 ± 0.73 |
| Orientation | 17.69 ± 0.92 |
| Abstract thinking | 9.24 ± 1.99 |
| Language | 9.73 ± 0.48 |
| Spatial construction | 9.84 ± 0.63 |
| Name fluency | 8.07 ± 2.15 |
| Mini–Mental State Examination | 26.91 ±1.69 |
| Center for Epidemiological Studies Depression | 11.33 ± 6.96 |

**Supplementary Figure. 1.** *In silico* data predicting the blood-brain barrier penetrative effect of indoxyl sulfate and p-cresyl sulfate


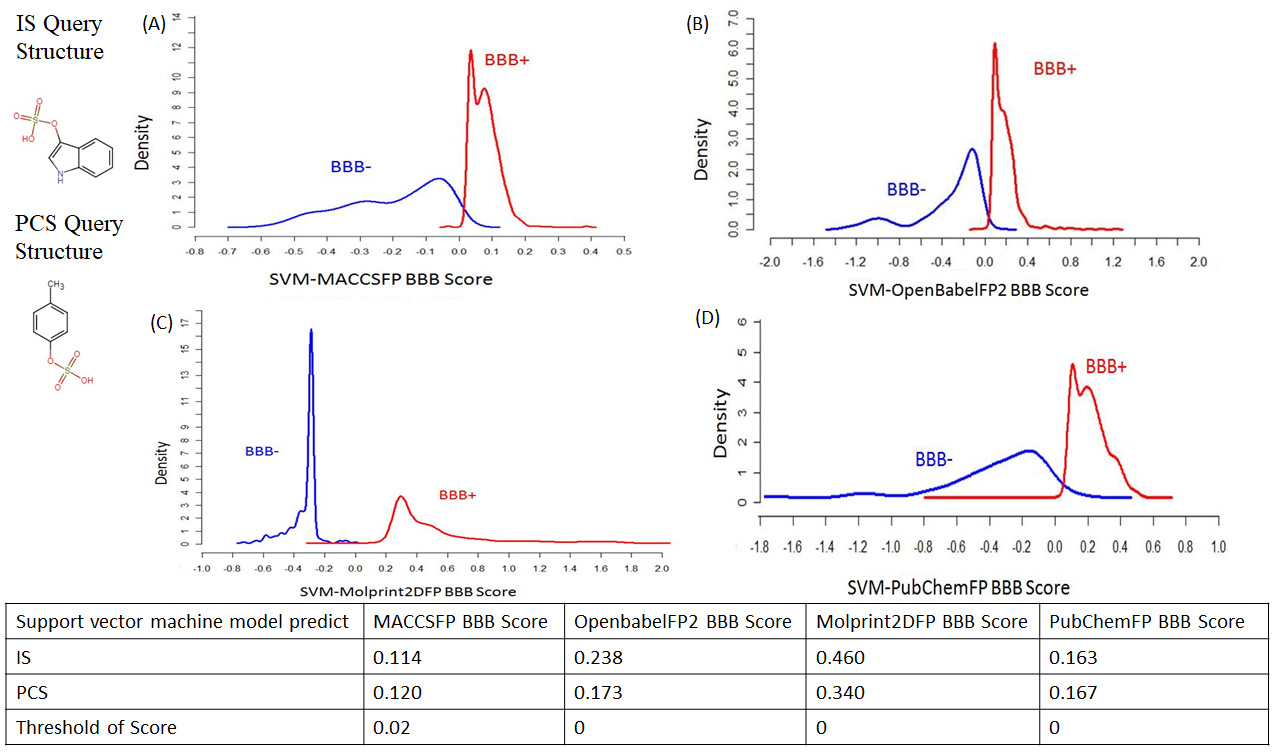

Supplement: Supplementary file 1 — Supplementary Tables and figures. [file 41598_2019_57004_MOESM1_ESM.docx]
